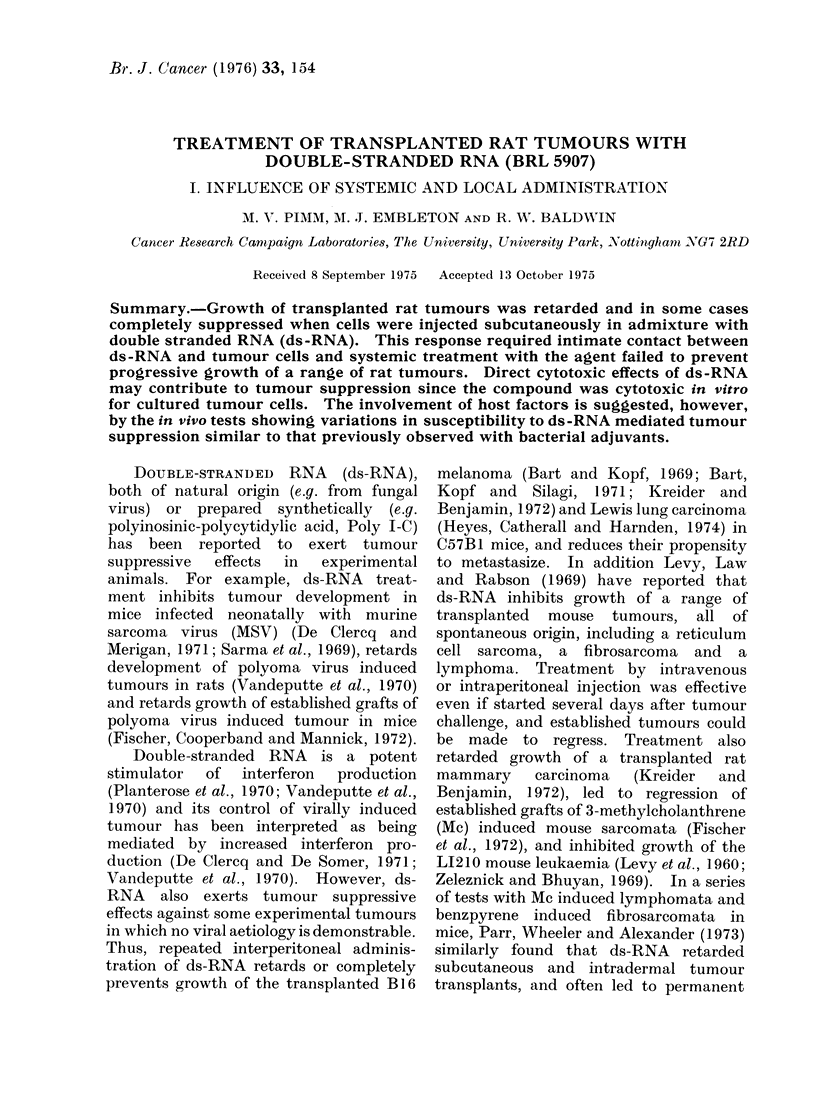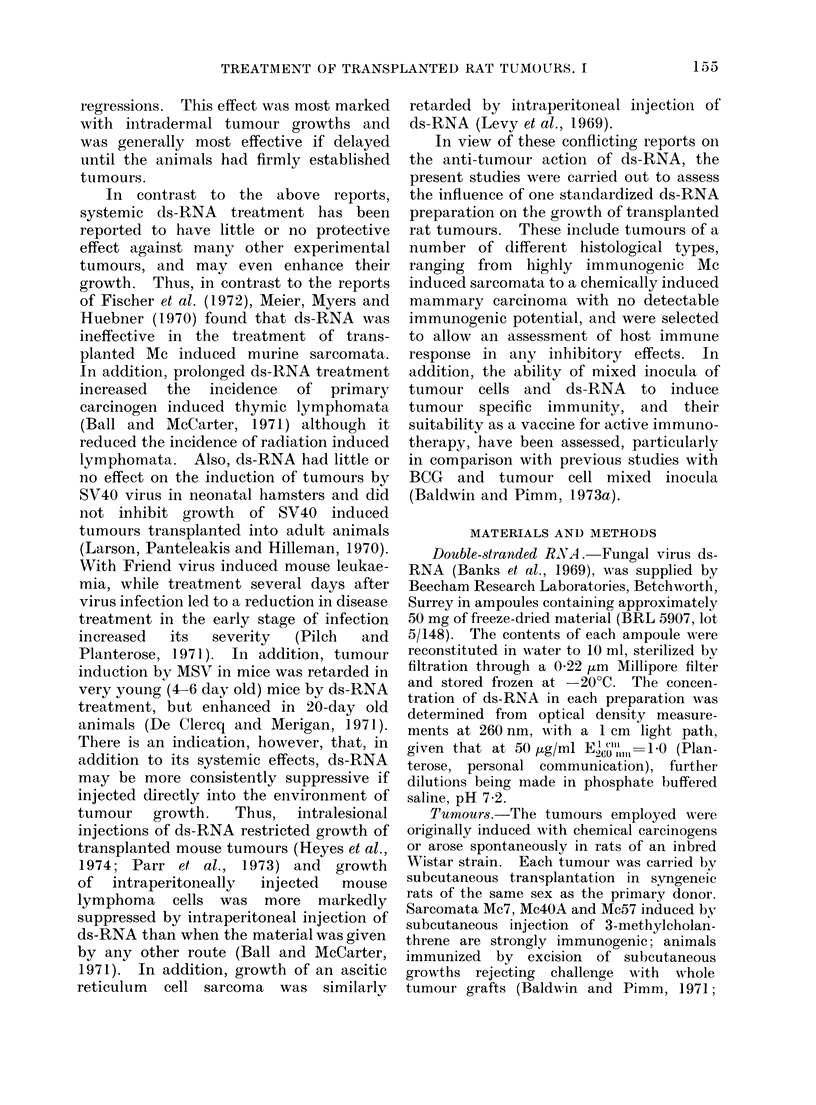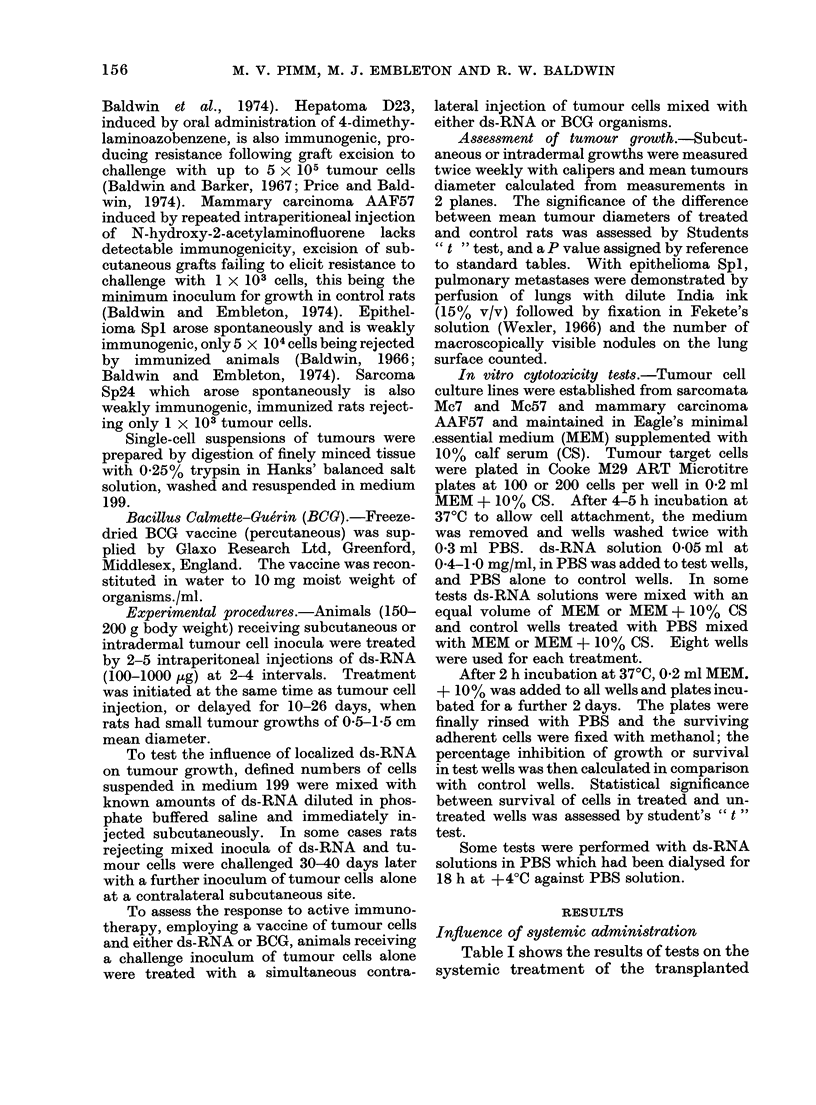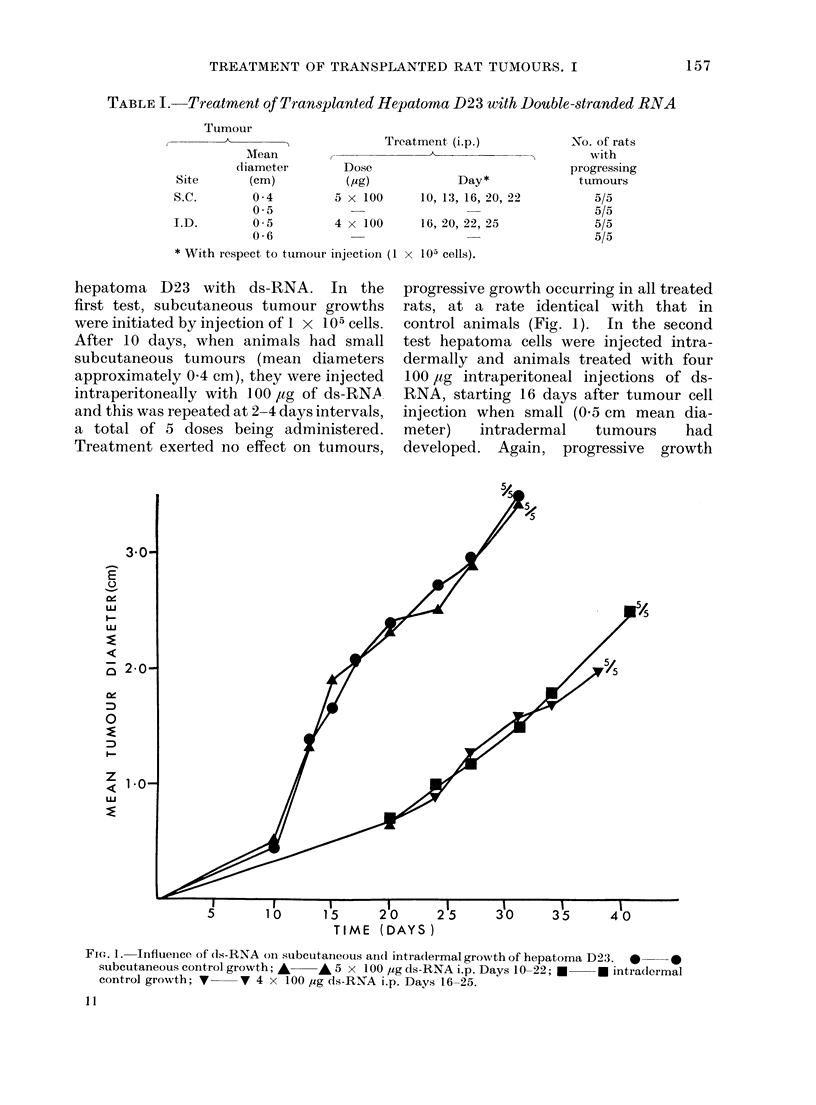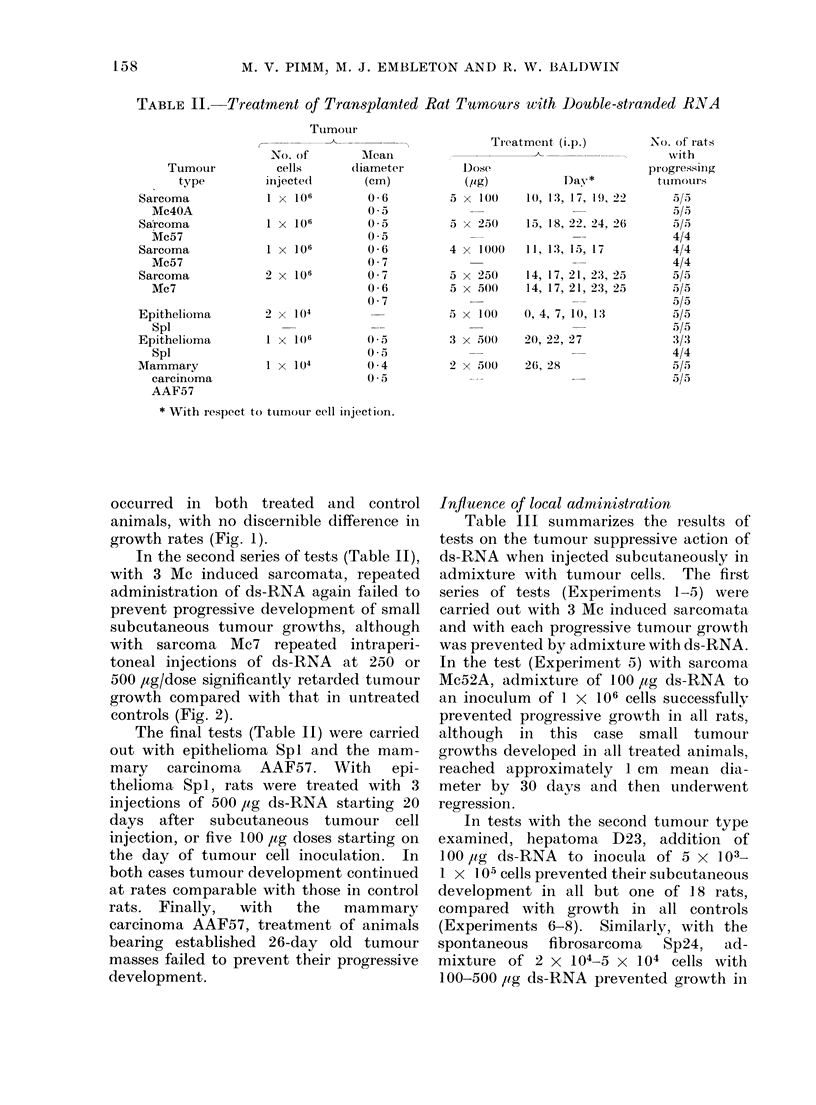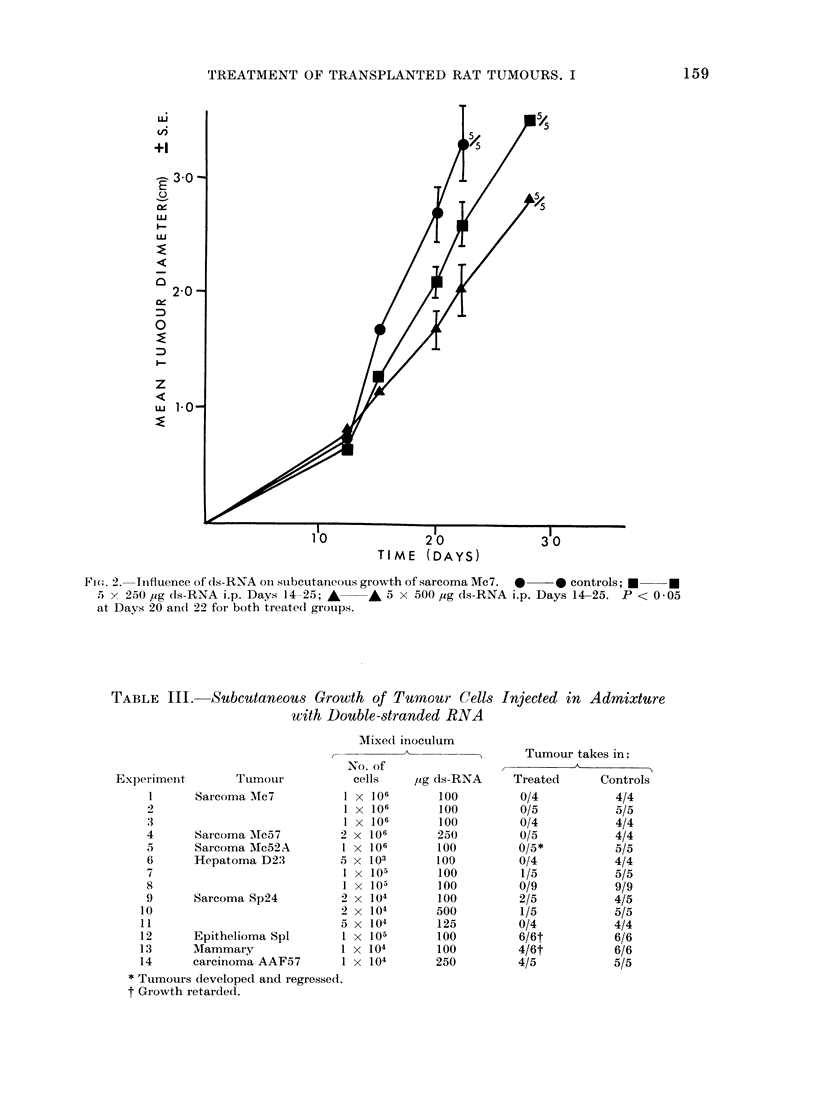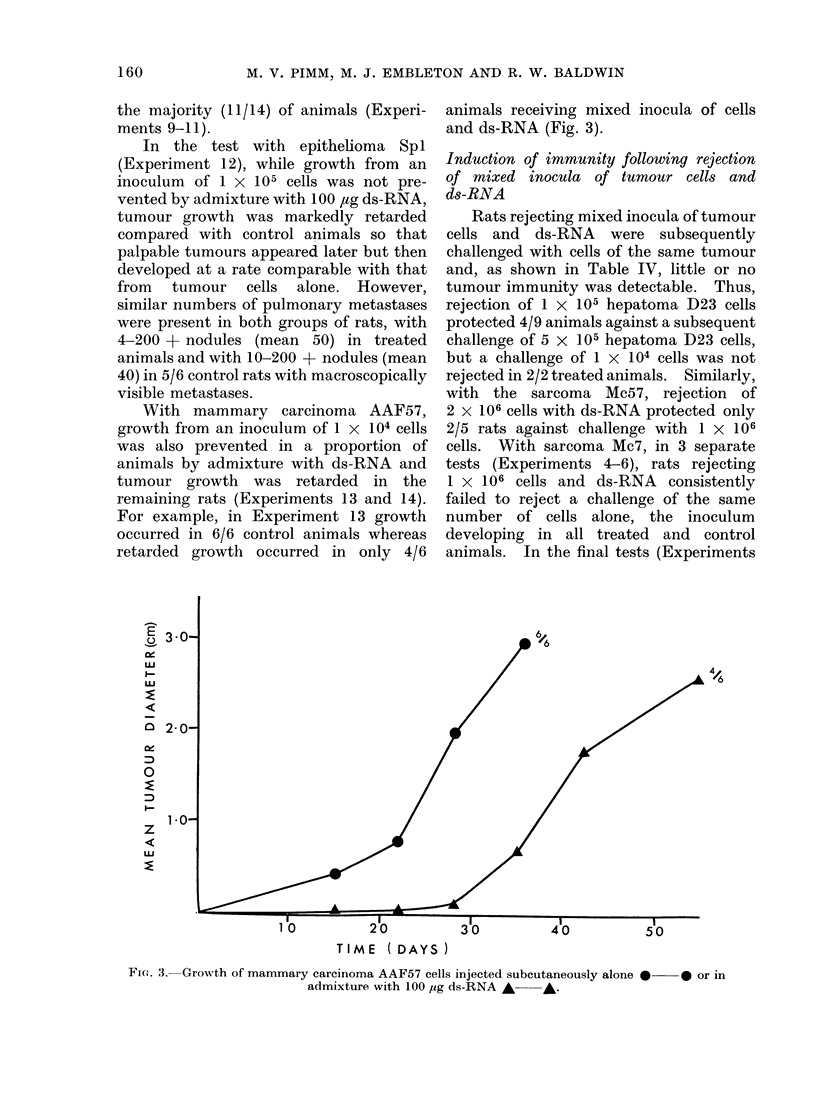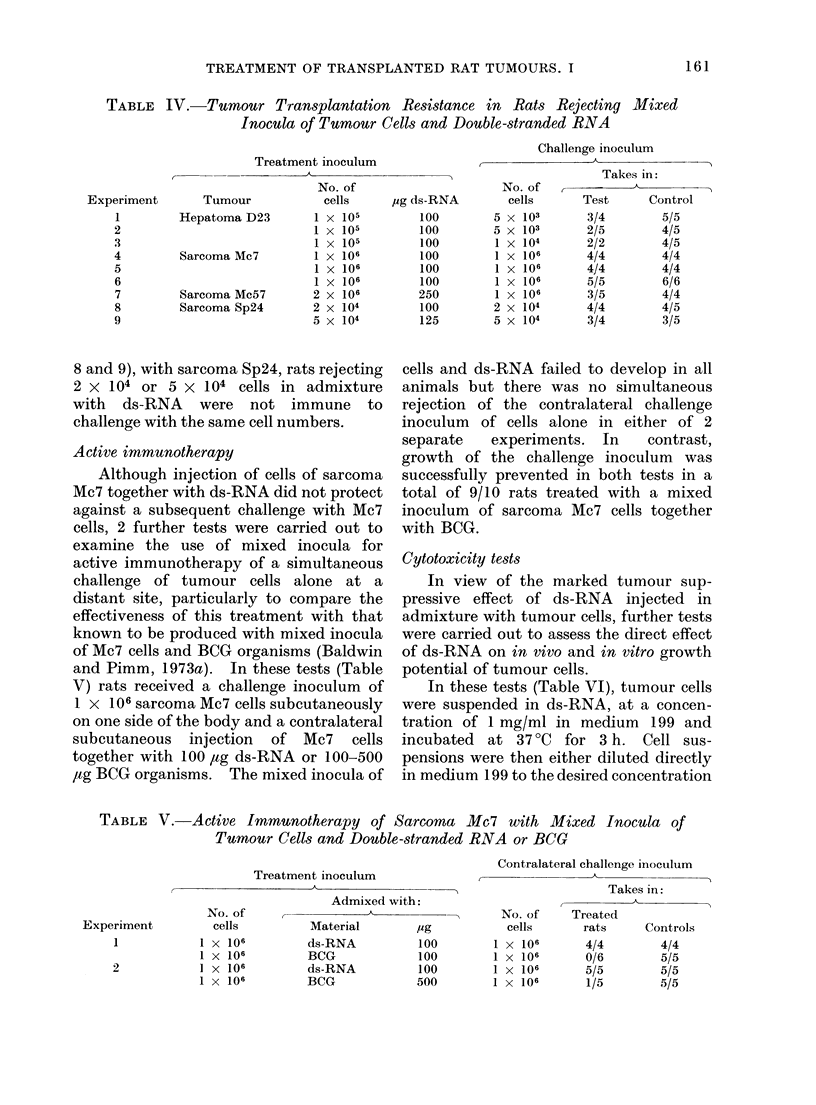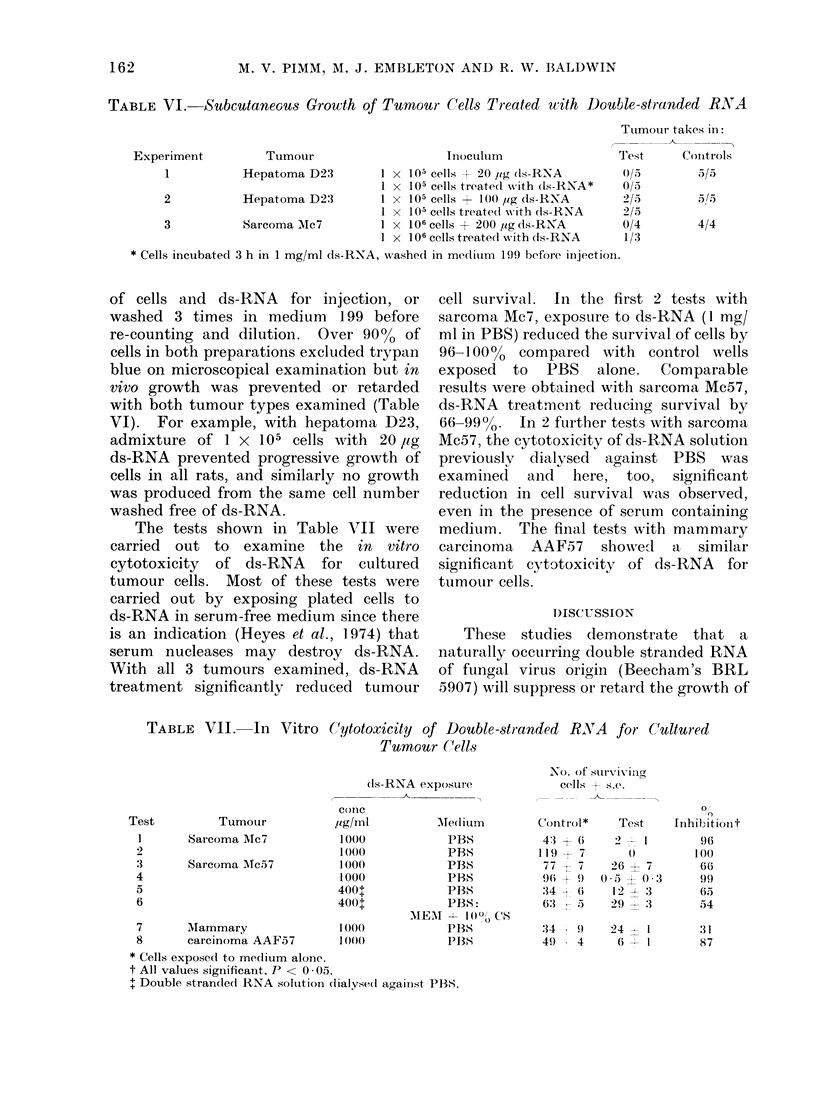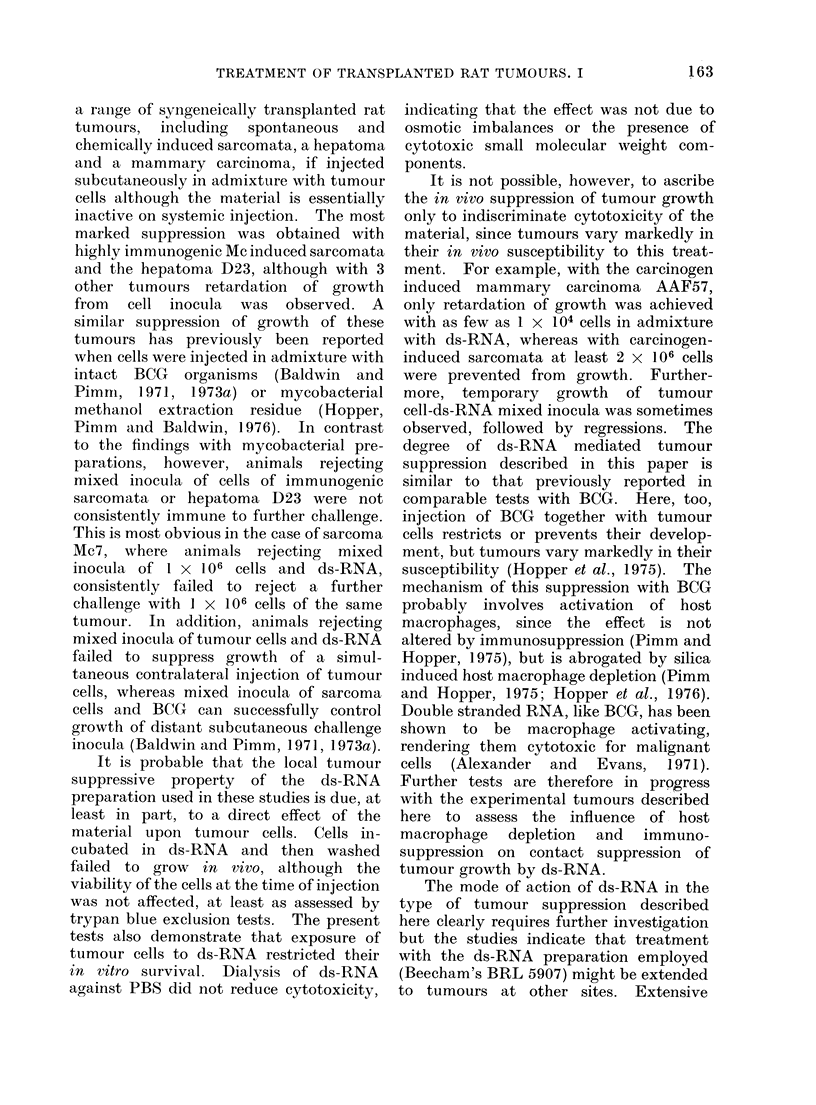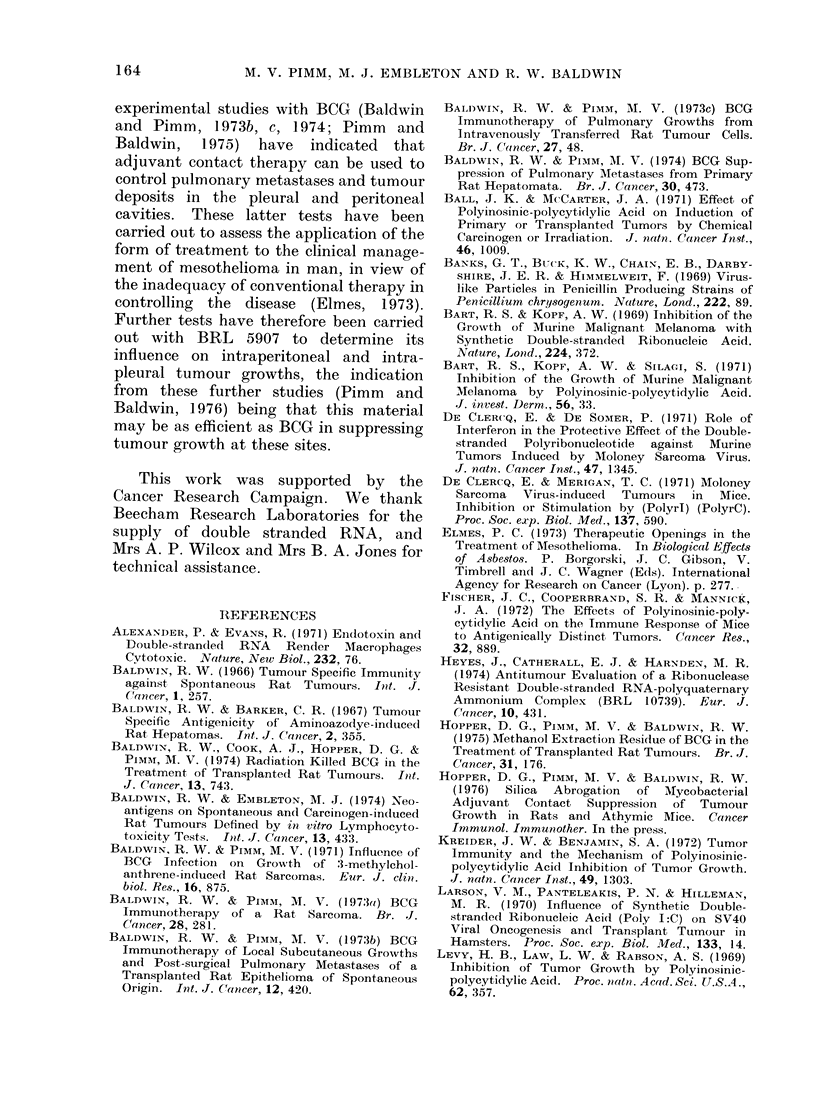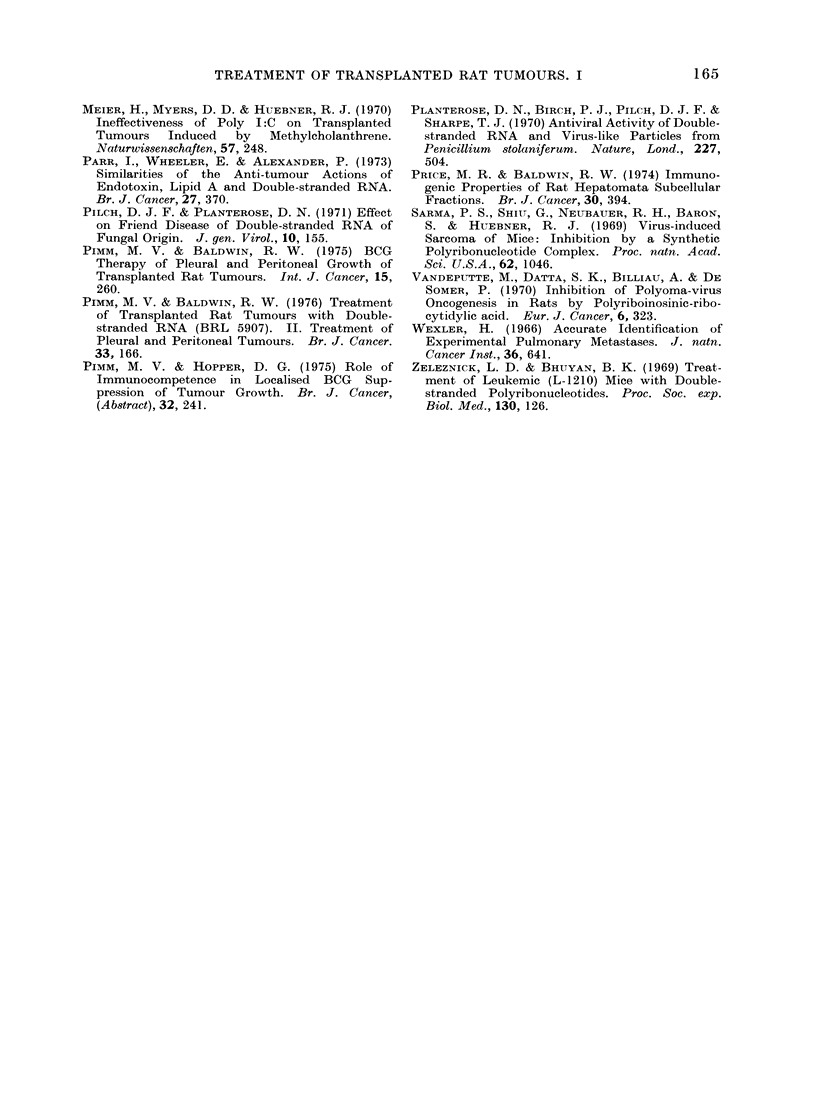# Treatment of transplanted rat tumours with double-stranded RNA (BRL 5907). I. Influenced of systemic and local administration.

**DOI:** 10.1038/bjc.1976.20

**Published:** 1976-02

**Authors:** M. V. Pimm, M. J. Embleton, R. W. Baldwin

## Abstract

Growth of transplanted rat tumours was retarded and in some cases completely suppressed when cells were injected subcutaneously in admixture with double stranded RNA (ds-RNA). This response required intimate contact between ds-RNA and tumour cells and systemic treatment with the agent failed to prevent progressive growth of a range of rat tumours. Direct cytotoxic effects of ds-RNA may contribute to tumour suppression since the compound was cytotoxic in vitro for cultured tumour cells. The involvement of host factors is suggested, however, by the in vivo tests showing variations in susceptibility to ds-RNA mediated tumour suppression similar to that previously observed with bacterial adjuvants.


*Br. J. Cancer* (1976) **33**, 154

### TREATMENT OF TRANSPLANTED RAT TUMOURS WITH DOUBLE-STRANDED RNA (BRL 5907)

### I. INFLUENCE OF SYSTEMIC AND LOCAL ADMINISTRATION

M. V. PIMM, M. J. EMBLETON AND R. W. BALDWIN

*Cancer Research Campaign Laboratories, The University, University Park, Nottingham NG7 2RD*

Received 8 September 1975 Accepted 13 October 1975

**Summary.—Growth of transplanted rat tumours was retarded and in some cases completely suppressed when cells were injected subcutaneously in admixture with double stranded RNA (ds-RNA). This response required intimate contact between ds-RNA and tumour cells and systemic treatment with the agent failed to prevent progressive growth of a range of rat tumours. Direct cytotoxic effects of ds-RNA may contribute to tumour suppression since the compound was cytotoxic *in vitro* for cultured tumour cells. The involvement of host factors is suggested, however, by the *in vivo* tests showing variations in susceptibility to ds-RNA mediated tumour suppression similar to that previously observed with bacterial adjuvants.**

DOUBLE-STRANDED RNA (ds-RNA), both of natural origin (*e.g.* from fungal virus) or prepared synthetically (*e.g.* polyinosinic-polycytidylic acid, Poly I-C) has been reported to exert tumour suppressive effects in experimental animals. For example, ds-RNA treatment inhibits tumour development in mice infected neonatally with murine sarcoma virus (MSV) (De Clercq and Merigan, 1971; Sarma *et al.*, 1969), retards development of polyoma virus induced tumours in rats (Vandeputte *et al.*, 1970) and retards growth of established grafts of polyoma virus induced tumour in mice (Fischer, Cooperband and Mannick, 1972).

Double-stranded RNA is a potent stimulator of interferon production (Planterose *et al.*, 1970; Vandeputte *et al.*, 1970) and its control of virally induced tumour has been interpreted as being mediated by increased interferon production (De Clercq and De Somer, 1971; Vandeputte *et al.*, 1970). However, ds-RNA also exerts tumour suppressive effects against some experimental tumours in which no viral aetiology is demonstrable. Thus, repeated interperitoneal administration of ds-RNA retards or completely prevents growth of the transplanted B16 melanoma (Bart and Kopf, 1969; Bart, Kopf and Silagi, 1971; Kreider and Benjamin, 1972) and Lewis lung carcinoma (Heyes, Catherall and Harnden, 1974) in C57B1 mice, and reduces their propensity to metastasize. In addition Levy, Law and Rabson (1969) have reported that ds-RNA inhibits growth of a range of transplanted mouse tumours, all of spontaneous origin, including a reticulum cell sarcoma, a fibrosarcoma and a lymphoma. Treatment by intravenous or intraperitoneal injection was effective even if started several days after tumour challenge, and established tumours could be made to regress. Treatment also retarded growth of a transplanted rat mammary carcinoma (Kreider and Benjamin, 1972), led to regression of established grafts of 3-methylcholanthrene (Mc) induced mouse sarcomata (Fischer *et al.*, 1972), and inhibited growth of the LI210 mouse leukaemia (Levy *et al.*, 1960; Zeleznick and Bhuyan, 1969). In a series of tests with Mc induced lymphomata and benzpyrene induced fibrosarcomata in mice, Parr, Wheeler and Alexander (1973) similarly found that ds-RNA retarded subcutaneous and intradermal tumour transplants, and often led to permanent regressions. This effect was most marked with intradermal tumour growths and was generally most effective if delayed until the animals had firmly established tumours.

In contrast to the above reports, systemic ds-RNA treatment has been reported to have little or no protective effect against many other experimental tumours, and may even enhance their growth. Thus, in contrast to the reports of Fischer *et al.* (1972), Meier, Myers and Huebner (1970) found that ds-RNA was ineffective in the treatment of transplanted Mc induced murine sarcomata. In addition, prolonged ds-RNA treatment increased the incidence of primary carcinogen induced thymic lymphomata (Ball and McCarter, 1971) although it reduced the incidence of radiation induced lymphomata. Also, ds-RNA had little or no effect on the induction of tumours by SV40 virus in neonatal hamsters and did not inhibit growth of SV40 induced tumours transplanted into adult animals (Larson, Panteleakis and Hilleman, 1970). With Friend virus induced mouse leukaemia, while treatment several days after virus infection led to a reduction in disease treatment in the early stage of infection increased its severity (Pilch and Planterose, 1971). In addition, tumour induction by MSV in mice was retarded in very young (4–6 day old) mice by ds-RNA treatment, but enhanced in 20-day old animals (De Clercq and Merigan, 1971). There is an indication, however, that, in addition to its systemic effects, ds-RNA may be more consistently suppressive if injected directly into the environment of tumour growth. Thus, intralesional injections of ds-RNA restricted growth of transplanted mouse tumours (Heyes *et al.*, 1974; Parr *et al.*, 1973) and growth of intraperitoneally injected mouse lymphoma cells was more markedly suppressed by intraperitoneal injection of ds-RNA than when the material was given by any other route (Ball and McCarter, 1971). In addition, growth of an ascitic reticulum cell sarcoma was similarly retarded by intraperitoneal injection of ds-RNA (Levy *et al.*, 1969).

In view of these conflicting reports on the anti-tumour action of ds-RNA, the present studies were carried out to assess the influence of one standardized ds-RNA preparation on the growth of transplanted rat tumours. These include tumours of a number of different histological types, ranging from highly immunogenic Mc induced sarcomata to a chemically induced mammary carcinoma with no detectable immunogenic potential, and were selected to allow an assessment of host immune response in any inhibitory effects. In addition, the ability of mixed inocula of tumour cells and ds-RNA to induce tumour specific immunity, and their suitability as a vaccine for active immunotherapy, have been assessed, particularly in comparison with previous studies with BCG and tumour cell mixed inocula (Baldwin and Pimm, 1973*a*).

### MATERIALS AND METHODS

*Double-stranded RNA*.—Fungal virus ds-RNA (Banks *et al.*, 1969), was supplied by Beecham Research Laboratories, Betchworth, Surrey in ampoules containing approximately 50 mg of freeze-dried material (BRL 5907, lot 5/148). The contents of each ampoule were reconstituted in water to 10 ml, sterilized by filtration through a 0·22 $\mu$m Millipore filter and stored frozen at $-20°C$. The concentration of ds-RNA in each preparation was determined from optical density measurements at 260 nm, with a 1 cm light path, given that at 50 $\mu$g/ml $E^{1\,cm}_{260\,nm} = 1{\cdot}0$ (Planterose, personal communication), further dilutions being made in phosphate buffered saline, pH 7·2.

*Tumours*.—The tumours employed were originally induced with chemical carcinogens or arose spontaneously in rats of an inbred Wistar strain. Each tumour was carried by subcutaneous transplantation in syngeneic rats of the same sex as the primary donor. Sarcomata Mc7, Mc40A and Mc57 induced by subcutaneous injection of 3-methylcholanthrene are strongly immunogenic; animals immunized by excision of subcutaneous growths rejecting challenge with whole tumour grafts (Baldwin and Pimm, 1971;

Baldwin *et al.*, 1974). Hepatoma D23, induced by oral administration of 4-dimethylaminoazobenzene, is also immunogenic, producing resistance following graft excision to challenge with up to $5 \times 10^5$ tumour cells (Baldwin and Barker, 1967; Price and Baldwin, 1974). Mammary carcinoma AAF57 induced by repeated intraperitioneal injection of N-hydroxy-2-acetylaminofluorene lacks detectable immunogenicity, excision of subcutaneous grafts failing to elicit resistance to challenge with $1 \times 10^3$ cells, this being the minimum inoculum for growth in control rats (Baldwin and Embleton, 1974). Epithelioma Spl arose spontaneously and is weakly immunogenic, only $5 \times 10^4$ cells being rejected by immunized animals (Baldwin, 1966; Baldwin and Embleton, 1974). Sarcoma Sp24 which arose spontaneously is also weakly immunogenic, immunized rats rejecting only $1 \times 10^3$ tumour cells.

Single-cell suspensions of tumours were prepared by digestion of finely minced tissue with 0·25% trypsin in Hanks' balanced salt solution, washed and resuspended in medium 199.

*Bacillus Calmette–Guérin (BCG)*.—Freeze-dried BCG vaccine (percutaneous) was supplied by Glaxo Research Ltd, Greenford, Middlesex, England. The vaccine was reconstituted in water to 10 mg moist weight of organisms./ml.

*Experimental procedures*.—Animals (150–200 g body weight) receiving subcutaneous or intradermal tumour cell inocula were treated by 2–5 intraperitoneal injections of ds-RNA (100–1000 μg) at 2–4 intervals. Treatment was initiated at the same time as tumour cell injection, or delayed for 10–26 days, when rats had small tumour growths of 0·5–1·5 cm mean diameter.

To test the influence of localized ds-RNA on tumour growth, defined numbers of cells suspended in medium 199 were mixed with known amounts of ds-RNA diluted in phosphate buffered saline and immediately injected subcutaneously. In some cases rats rejecting mixed inocula of ds-RNA and tumour cells were challenged 30–40 days later with a further inoculum of tumour cells alone at a contralateral subcutaneous site.

To assess the response to active immunotherapy, employing a vaccine of tumour cells and either ds-RNA or BCG, animals receiving a challenge inoculum of tumour cells alone were treated with a simultaneous contralateral injection of tumour cells mixed with either ds-RNA or BCG organisms.

*Assessment of tumour growth*.—Subcutaneous or intradermal growths were measured twice weekly with calipers and mean tumours diameter calculated from measurements in 2 planes. The significance of the difference between mean tumour diameters of treated and control rats was assessed by Students " *t* " test, and a *P* value assigned by reference to standard tables. With epithelioma Spl, pulmonary metastases were demonstrated by perfusion of lungs with dilute India ink (15% v/v) followed by fixation in Fekete's solution (Wexler, 1966) and the number of macroscopically visible nodules on the lung surface counted.

*In vitro cytotoxicity tests*.—Tumour cell culture lines were established from sarcomata Mc7 and Mc57 and mammary carcinoma AAF57 and maintained in Eagle's minimal essential medium (MEM) supplemented with 10% calf serum (CS). Tumour target cells were plated in Cooke M29 ART Microtitre plates at 100 or 200 cells per well in 0·2 ml MEM + 10% CS. After 4–5 h incubation at 37°C to allow cell attachment, the medium was removed and wells washed twice with 0·3 ml PBS. ds-RNA solution 0·05 ml at 0·4–1·0 mg/ml, in PBS was added to test wells, and PBS alone to control wells. In some tests ds-RNA solutions were mixed with an equal volume of MEM or MEM + 10% CS and control wells treated with PBS mixed with MEM or MEM + 10% CS. Eight wells were used for each treatment.

After 2 h incubation at 37°C, 0·2 ml MEM. + 10% was added to all wells and plates incubated for a further 2 days. The plates were finally rinsed with PBS and the surviving adherent cells were fixed with methanol; the percentage inhibition of growth or survival in test wells was then calculated in comparison with control wells. Statistical significance between survival of cells in treated and untreated wells was assessed by student's " *t* " test.

Some tests were performed with ds-RNA solutions in PBS which had been dialysed for 18 h at +4°C against PBS solution.

### RESULTS

### *Influence of systemic administration*

Table I shows the results of tests on the systemic treatment of the transplanted

TABLE I.—*Treatment of Transplanted Hepatoma D23 with Double-stranded RNA*

| Tumour | | Treatment (i.p.) | | No. of rats with progressing tumours |
|---|---|---|---|---|
| Site | Mean diameter (cm) | Dose ($\mu$g) | Day* | |
| S.C. | 0·4 | 5 × 100 | 10, 13, 16, 20, 22 | 5/5 |
| | 0·5 | — | — | 5/5 |
| I.D. | 0·5 | 4 × 100 | 16, 20, 22, 25 | 5/5 |
| | 0·6 | — | — | 5/5 |

* With respect to tumour injection (1 × $10^5$ cells).

hepatoma D23 with ds-RNA. In the first test, subcutaneous tumour growths were initiated by injection of 1 × $10^5$ cells. After 10 days, when animals had small subcutaneous tumours (mean diameters approximately 0·4 cm), they were injected intraperitoneally with 100 $\mu$g of ds-RNA and this was repeated at 2–4 days intervals, a total of 5 doses being administered. Treatment exerted no effect on tumours, progressive growth occurring in all treated rats, at a rate identical with that in control animals (Fig. 1). In the second test hepatoma cells were injected intradermally and animals treated with four 100 $\mu$g intraperitoneal injections of ds-RNA, starting 16 days after tumour cell injection when small (0·5 cm mean diameter) intradermal tumours had developed. Again, progressive growth

FIG. 1.—Influence of ds-RNA on subcutaneous and intradermal growth of hepatoma D23. ●——● subcutaneous control growth; ▲——▲ 5 × 100 $\mu$g ds-RNA i.p. Days 10–22; ■——■ intradermal control growth; ▼——▼ 4 × 100 $\mu$g ds-RNA i.p. Days 16–25.

TABLE II.—*Treatment of Transplanted Rat Tumours with Double-stranded RNA*

| Tumour type | Tumour: No. of cells injected | Tumour: Mean diameter (cm) | Treatment (i.p.): Dose (μg) | Treatment (i.p.): Day* | No. of rats with progressing tumours |
|---|---|---|---|---|---|
| Sarcoma | 1 × $10^6$ | 0·6 | 5 × 100 | 10, 13, 17, 19, 22 | 5/5 |
| Mc40A | | 0·5 | — | — | 5/5 |
| Sarcoma | 1 × $10^6$ | 0·5 | 5 × 250 | 15, 18, 22, 24, 26 | 5/5 |
| Mc57 | | 0·5 | — | — | 4/4 |
| Sarcoma | 1 × $10^6$ | 0·6 | 4 × 1000 | 11, 13, 15, 17 | 4/4 |
| Mc57 | | 0·7 | — | — | 4/4 |
| Sarcoma | 2 × $10^6$ | 0·7 | 5 × 250 | 14, 17, 21, 23, 25 | 5/5 |
| Mc7 | | 0·6 | 5 × 500 | 14, 17, 21, 23, 25 | 5/5 |
| | | 0·7 | — | — | 5/5 |
| Epithelioma | 2 × $10^4$ | — | 5 × 100 | 0, 4, 7, 10, 13 | 5/5 |
| Spl | — | — | — | — | 5/5 |
| Epithelioma | 1 × $10^6$ | 0·5 | 3 × 500 | 20, 22, 27 | 3/3 |
| Spl | | 0·5 | — | — | 4/4 |
| Mammary | 1 × $10^4$ | 0·4 | 2 × 500 | 26, 28 | 5/5 |
| carcinoma | | 0·5 | — | — | 5/5 |
| AAF57 | | | | | |

* With respect to tumour cell injection.

occurred in both treated and control animals, with no discernible difference in growth rates (Fig. 1).

In the second series of tests (Table II), with 3 Mc induced sarcomata, repeated administration of ds-RNA again failed to prevent progressive development of small subcutaneous tumour growths, although with sarcoma Mc7 repeated intraperitoneal injections of ds-RNA at 250 or 500 μg/dose significantly retarded tumour growth compared with that in untreated controls (Fig. 2).

The final tests (Table II) were carried out with epithelioma Spl and the mammary carcinoma AAF57. With epithelioma Spl, rats were treated with 3 injections of 500 μg ds-RNA starting 20 days after subcutaneous tumour cell injection, or five 100 μg doses starting on the day of tumour cell inoculation. In both cases tumour development continued at rates comparable with those in control rats. Finally, with the mammary carcinoma AAF57, treatment of animals bearing established 26-day old tumour masses failed to prevent their progressive development.

*Influence of local administration*

Table III summarizes the results of tests on the tumour suppressive action of ds-RNA when injected subcutaneously in admixture with tumour cells. The first series of tests (Experiments 1–5) were carried out with 3 Mc induced sarcomata and with each progressive tumour growth was prevented by admixture with ds-RNA. In the test (Experiment 5) with sarcoma Mc52A, admixture of 100 μg ds-RNA to an inoculum of 1 × $10^6$ cells successfully prevented progressive growth in all rats, although in this case small tumour growths developed in all treated animals, reached approximately 1 cm mean diameter by 30 days and then underwent regression.

In tests with the second tumour type examined, hepatoma D23, addition of 100 μg ds-RNA to inocula of 5 × $10^3$–1 × $10^5$ cells prevented their subcutaneous development in all but one of 18 rats, compared with growth in all controls (Experiments 6–8). Similarly, with the spontaneous fibrosarcoma Sp24, admixture of 2 × $10^4$–5 × $10^4$ cells with 100–500 μg ds-RNA prevented growth in

FIG. 2.—Influence of ds-RNA on subcutaneous growth of sarcoma Mc7. ●——● controls; ■——■ 5 × 250 $\mu g$ ds-RNA i.p. Days 14–25; ▲——▲ 5 × 500 $\mu g$ ds-RNA i.p. Days 14–25. $P < 0 \cdot 05$ at Days 20 and 22 for both treated groups.

TABLE III.—*Subcutaneous Growth of Tumour Cells Injected in Admixture with Double-stranded RNA*

| | | Mixed inoculum | | Tumour takes in: | |
|---|---|---|---|---|---|
| Experiment | Tumour | No. of cells | $\mu g$ ds-RNA | Treated | Controls |
| 1 | Sarcoma Mc7 | $1 \times 10^6$ | 100 | 0/4 | 4/4 |
| 2 | | $1 \times 10^6$ | 100 | 0/5 | 5/5 |
| 3 | | $1 \times 10^6$ | 100 | 0/4 | 4/4 |
| 4 | Sarcoma Mc57 | $2 \times 10^6$ | 250 | 0/5 | 4/4 |
| 5 | Sarcoma Mc52A | $1 \times 10^6$ | 100 | 0/5* | 5/5 |
| 6 | Hepatoma D23 | $5 \times 10^3$ | 100 | 0/4 | 4/4 |
| 7 | | $1 \times 10^5$ | 100 | 1/5 | 5/5 |
| 8 | | $1 \times 10^5$ | 100 | 0/9 | 9/9 |
| 9 | Sarcoma Sp24 | $2 \times 10^4$ | 100 | 2/5 | 4/5 |
| 10 | | $2 \times 10^4$ | 500 | 1/5 | 5/5 |
| 11 | | $5 \times 10^4$ | 125 | 0/4 | 4/4 |
| 12 | Epithelioma Spl | $1 \times 10^5$ | 100 | 6/6† | 6/6 |
| 13 | Mammary | $1 \times 10^4$ | 100 | 4/6† | 6/6 |
| 14 | carcinoma AAF57 | $1 \times 10^4$ | 250 | 4/5 | 5/5 |

* Tumours developed and regressed.
† Growth retarded.

the majority (11/14) of animals (Experiments 9–11).

In the test with epithelioma Spl (Experiment 12), while growth from an inoculum of $1 \times 10^5$ cells was not prevented by admixture with 100 $\mu$g ds-RNA, tumour growth was markedly retarded compared with control animals so that palpable tumours appeared later but then developed at a rate comparable with that from tumour cells alone. However, similar numbers of pulmonary metastases were present in both groups of rats, with 4–200 + nodules (mean 50) in treated animals and with 10–200 + nodules (mean 40) in 5/6 control rats with macroscopically visible metastases.

With mammary carcinoma AAF57, growth from an inoculum of $1 \times 10^4$ cells was also prevented in a proportion of animals by admixture with ds-RNA and tumour growth was retarded in the remaining rats (Experiments 13 and 14). For example, in Experiment 13 growth occurred in 6/6 control animals whereas retarded growth occurred in only 4/6 animals receiving mixed inocula of cells and ds-RNA (Fig. 3).

*Induction of immunity following rejection of mixed inocula of tumour cells and ds-RNA*

Rats rejecting mixed inocula of tumour cells and ds-RNA were subsequently challenged with cells of the same tumour and, as shown in Table IV, little or no tumour immunity was detectable. Thus, rejection of $1 \times 10^5$ hepatoma D23 cells protected 4/9 animals against a subsequent challenge of $5 \times 10^5$ hepatoma D23 cells, but a challenge of $1 \times 10^4$ cells was not rejected in 2/2 treated animals. Similarly, with the sarcoma Mc57, rejection of $2 \times 10^6$ cells with ds-RNA protected only 2/5 rats against challenge with $1 \times 10^6$ cells. With sarcoma Mc7, in 3 separate tests (Experiments 4–6), rats rejecting $1 \times 10^6$ cells and ds-RNA consistently failed to reject a challenge of the same number of cells alone, the inoculum developing in all treated and control animals. In the final tests (Experiments

FIG. 3.—Growth of mammary carcinoma AAF57 cells injected subcutaneously alone ●——● or in admixture with 100 $\mu$g ds-RNA ▲——▲.

TABLE IV.—*Tumour Transplantation Resistance in Rats Rejecting Mixed Inocula of Tumour Cells and Double-stranded RNA*

| | Treatment inoculum | | | Challenge inoculum | | |
|---|---|---|---|---|---|---|
| | | | | | Takes in: | |
| Experiment | Tumour | No. of cells | $\mu g$ ds-RNA | No. of cells | Test | Control |
| 1 | Hepatoma D23 | $1 \times 10^5$ | 100 | $5 \times 10^3$ | 3/4 | 5/5 |
| 2 | | $1 \times 10^5$ | 100 | $5 \times 10^3$ | 2/5 | 4/5 |
| 3 | | $1 \times 10^5$ | 100 | $1 \times 10^4$ | 2/2 | 4/5 |
| 4 | Sarcoma Mc7 | $1 \times 10^6$ | 100 | $1 \times 10^6$ | 4/4 | 4/4 |
| 5 | | $1 \times 10^6$ | 100 | $1 \times 10^6$ | 4/4 | 4/4 |
| 6 | | $1 \times 10^6$ | 100 | $1 \times 10^6$ | 5/5 | 6/6 |
| 7 | Sarcoma Mc57 | $2 \times 10^6$ | 250 | $1 \times 10^6$ | 3/5 | 4/4 |
| 8 | Sarcoma Sp24 | $2 \times 10^4$ | 100 | $2 \times 10^4$ | 4/4 | 4/5 |
| 9 | | $5 \times 10^4$ | 125 | $5 \times 10^4$ | 3/4 | 3/5 |

8 and 9), with sarcoma Sp24, rats rejecting $2 \times 10^4$ or $5 \times 10^4$ cells in admixture with ds-RNA were not immune to challenge with the same cell numbers.

### *Active immunotherapy*

Although injection of cells of sarcoma Mc7 together with ds-RNA did not protect against a subsequent challenge with Mc7 cells, 2 further tests were carried out to examine the use of mixed inocula for active immunotherapy of a simultaneous challenge of tumour cells alone at a distant site, particularly to compare the effectiveness of this treatment with that known to be produced with mixed inocula of Mc7 cells and BCG organisms (Baldwin and Pimm, 1973*a*). In these tests (Table V) rats received a challenge inoculum of $1 \times 10^6$ sarcoma Mc7 cells subcutaneously on one side of the body and a contralateral subcutaneous injection of Mc7 cells together with 100 $\mu g$ ds-RNA or 100–500 $\mu g$ BCG organisms. The mixed inocula of cells and ds-RNA failed to develop in all animals but there was no simultaneous rejection of the contralateral challenge inoculum of cells alone in either of 2 separate experiments. In contrast, growth of the challenge inoculum was successfully prevented in both tests in a total of 9/10 rats treated with a mixed inoculum of sarcoma Mc7 cells together with BCG.

### *Cytotoxicity tests*

In view of the marked tumour suppressive effect of ds-RNA injected in admixture with tumour cells, further tests were carried out to assess the direct effect of ds-RNA on *in vivo* and *in vitro* growth potential of tumour cells.

In these tests (Table VI), tumour cells were suspended in ds-RNA, at a concentration of 1 mg/ml in medium 199 and incubated at 37 °C for 3 h. Cell suspensions were then either diluted directly in medium 199 to the desired concentration

TABLE V.—*Active Immunotherapy of Sarcoma Mc7 with Mixed Inocula of Tumour Cells and Double-stranded RNA or BCG*

| | Treatment inoculum | | | Contralateral challenge inoculum | | |
|---|---|---|---|---|---|---|
| | | Admixed with: | | | Takes in: | |
| Experiment | No. of cells | Material | $\mu g$ | No. of cells | Treated rats | Controls |
| 1 | $1 \times 10^6$ | ds-RNA | 100 | $1 \times 10^6$ | 4/4 | 4/4 |
| | $1 \times 10^6$ | BCG | 100 | $1 \times 10^6$ | 0/6 | 5/5 |
| 2 | $1 \times 10^6$ | ds-RNA | 100 | $1 \times 10^6$ | 5/5 | 5/5 |
| | $1 \times 10^6$ | BCG | 500 | $1 \times 10^6$ | 1/5 | 5/5 |

TABLE VI.—*Subcutaneous Growth of Tumour Cells Treated with Double-stranded RNA*

| Experiment | Tumour | Inoculum | Tumour takes in: Test | Controls |
|---|---|---|---|---|
| 1 | Hepatoma D23 | $1 \times 10^5$ cells + 20 $\mu$g ds-RNA | 0/5 | 5/5 |
| | | $1 \times 10^5$ cells treated with ds-RNA* | 0/5 | |
| 2 | Hepatoma D23 | $1 \times 10^5$ cells + 100 $\mu$g ds-RNA | 2/5 | 5/5 |
| | | $1 \times 10^5$ cells treated with ds-RNA | 2/5 | |
| 3 | Sarcoma Mc7 | $1 \times 10^6$ cells + 200 $\mu$g ds-RNA | 0/4 | 4/4 |
| | | $1 \times 10^6$ cells treated with ds-RNA | 1/3 | |

* Cells incubated 3 h in 1 mg/ml ds-RNA, washed in medium 199 before injection.

of cells and ds-RNA for injection, or washed 3 times in medium 199 before re-counting and dilution. Over 90% of cells in both preparations excluded trypan blue on microscopical examination but *in vivo* growth was prevented or retarded with both tumour types examined (Table VI). For example, with hepatoma D23, admixture of $1 \times 10^5$ cells with 20 $\mu$g ds-RNA prevented progressive growth of cells in all rats, and similarly no growth was produced from the same cell number washed free of ds-RNA.

The tests shown in Table VII were carried out to examine the *in vitro* cytotoxicity of ds-RNA for cultured tumour cells. Most of these tests were carried out by exposing plated cells to ds-RNA in serum-free medium since there is an indication (Heyes *et al.*, 1974) that serum nucleases may destroy ds-RNA. With all 3 tumours examined, ds-RNA treatment significantly reduced tumour cell survival. In the first 2 tests with sarcoma Mc7, exposure to ds-RNA (1 mg/ml in PBS) reduced the survival of cells by 96–100% compared with control wells exposed to PBS alone. Comparable results were obtained with sarcoma Mc57, ds-RNA treatment reducing survival by 66–99%. In 2 further tests with sarcoma Mc57, the cytotoxicity of ds-RNA solution previously dialysed against PBS was examined and here, too, significant reduction in cell survival was observed, even in the presence of serum containing medium. The final tests with mammary carcinoma AAF57 showed a similar significant cytotoxicity of ds-RNA for tumour cells.

### DISCUSSION

These studies demonstrate that a naturally occurring double stranded RNA of fungal virus origin (Beecham's BRL 5907) will suppress or retard the growth of

TABLE VII.—In Vitro *Cytotoxicity of Double-stranded RNA for Cultured Tumour Cells*

| Test | Tumour | ds-RNA exposure: conc $\mu$g/ml | Medium | No. of surviving cells ± s.e.: Control* | Test | % Inhibition† |
|---|---|---|---|---|---|---|
| 1 | Sarcoma Mc7 | 1000 | PBS | 43 ± 6 | 2 ± 1 | 96 |
| 2 | | 1000 | PBS | 119 ± 7 | 0 | 100 |
| 3 | Sarcoma Mc57 | 1000 | PBS | 77 ± 7 | 26 ± 7 | 66 |
| 4 | | 1000 | PBS | 96 ± 9 | 0·5 ± 0·3 | 99 |
| 5 | | 400‡ | PBS | 34 ± 6 | 12 ± 3 | 65 |
| 6 | | 400‡ | PBS: MEM + 10% CS | 63 ± 5 | 29 ± 3 | 54 |
| 7 | Mammary | 1000 | PBS | 34 ± 9 | 24 ± 1 | 31 |
| 8 | carcinoma AAF57 | 1000 | PBS | 49 ± 4 | 6 ± 1 | 87 |

* Cells exposed to medium alone.

† All values significant, $P < 0 \cdot 05$.

‡ Double stranded RNA solution dialysed against PBS.

a range of syngeneically transplanted rat tumours, including spontaneous and chemically induced sarcomata, a hepatoma and a mammary carcinoma, if injected subcutaneously in admixture with tumour cells although the material is essentially inactive on systemic injection. The most marked suppression was obtained with highly immunogenic Mc induced sarcomata and the hepatoma D23, although with 3 other tumours retardation of growth from cell inocula was observed. A similar suppression of growth of these tumours has previously been reported when cells were injected in admixture with intact BCG organisms (Baldwin and Pimm, 1971, 1973*a*) or mycobacterial methanol extraction residue (Hopper, Pimm and Baldwin, 1976). In contrast to the findings with mycobacterial preparations, however, animals rejecting mixed inocula of cells of immunogenic sarcomata or hepatoma D23 were not consistently immune to further challenge. This is most obvious in the case of sarcoma Mc7, where animals rejecting mixed inocula of $1 \times 10^6$ cells and ds-RNA, consistently failed to reject a further challenge with $1 \times 10^6$ cells of the same tumour. In addition, animals rejecting mixed inocula of tumour cells and ds-RNA failed to suppress growth of a simultaneous contralateral injection of tumour cells, whereas mixed inocula of sarcoma cells and BCG can successfully control growth of distant subcutaneous challenge inocula (Baldwin and Pimm, 1971, 1973*a*).

It is probable that the local tumour suppressive property of the ds-RNA preparation used in these studies is due, at least in part, to a direct effect of the material upon tumour cells. Cells incubated in ds-RNA and then washed failed to grow *in vivo*, although the viability of the cells at the time of injection was not affected, at least as assessed by trypan blue exclusion tests. The present tests also demonstrate that exposure of tumour cells to ds-RNA restricted their *in vitro* survival. Dialysis of ds-RNA against PBS did not reduce cytotoxicity, indicating that the effect was not due to osmotic imbalances or the presence of cytotoxic small molecular weight components.

It is not possible, however, to ascribe the *in vivo* suppression of tumour growth only to indiscriminate cytotoxicity of the material, since tumours vary markedly in their *in vivo* susceptibility to this treatment. For example, with the carcinogen induced mammary carcinoma AAF57, only retardation of growth was achieved with as few as $1 \times 10^4$ cells in admixture with ds-RNA, whereas with carcinogen-induced sarcomata at least $2 \times 10^6$ cells were prevented from growth. Furthermore, temporary growth of tumour cell-ds-RNA mixed inocula was sometimes observed, followed by regressions. The degree of ds-RNA mediated tumour suppression described in this paper is similar to that previously reported in comparable tests with BCG. Here, too, injection of BCG together with tumour cells restricts or prevents their development, but tumours vary markedly in their susceptibility (Hopper *et al.*, 1975). The mechanism of this suppression with BCG probably involves activation of host macrophages, since the effect is not altered by immunosuppression (Pimm and Hopper, 1975), but is abrogated by silica induced host macrophage depletion (Pimm and Hopper, 1975; Hopper *et al.*, 1976). Double stranded RNA, like BCG, has been shown to be macrophage activating, rendering them cytotoxic for malignant cells (Alexander and Evans, 1971). Further tests are therefore in progress with the experimental tumours described here to assess the influence of host macrophage depletion and immunosuppression on contact suppression of tumour growth by ds-RNA.

The mode of action of ds-RNA in the type of tumour suppression described here clearly requires further investigation but the studies indicate that treatment with the ds-RNA preparation employed (Beecham's BRL 5907) might be extended to tumours at other sites. Extensive experimental studies with BCG (Baldwin and Pimm, 1973*b*, *c*, 1974; Pimm and Baldwin, 1975) have indicated that adjuvant contact therapy can be used to control pulmonary metastases and tumour deposits in the pleural and peritoneal cavities. These latter tests have been carried out to assess the application of the form of treatment to the clinical management of mesothelioma in man, in view of the inadequacy of conventional therapy in controlling the disease (Elmes, 1973). Further tests have therefore been carried out with BRL 5907 to determine its influence on intraperitoneal and intrapleural tumour growths, the indication from these further studies (Pimm and Baldwin, 1976) being that this material may be as efficient as BCG in suppressing tumour growth at these sites.

This work was supported by the Cancer Research Campaign. We thank Beecham Research Laboratories for the supply of double stranded RNA, and Mrs A. P. Wilcox and Mrs B. A. Jones for technical assistance.

### REFERENCES

ALEXANDER, P. & EVANS, R. (1971) Endotoxin and Double-stranded RNA Render Macrophages Cytotoxic. *Nature, New Biol.*, **232**, 76.

BALDWIN, R. W. (1966) Tumour Specific Immunity against Spontaneous Rat Tumours. *Int. J. Cancer*, **1**, 257.

BALDWIN, R. W. & BARKER, C. R. (1967) Tumour Specific Antigenicity of Aminoazodye-induced Rat Hepatomas. *Int. J. Cancer*, **2**, 355.

BALDWIN, R. W., COOK, A. J., HOPPER, D. G. & PIMM, M. V. (1974) Radiation Killed BCG in the Treatment of Transplanted Rat Tumours. *Int. J. Cancer*, **13**, 743.

BALDWIN, R. W. & EMBLETON, M. J. (1974) Neoantigens on Spontaneous and Carcinogen-induced Rat Tumours Defined by *in vitro* Lymphocytotoxicity Tests. *Int. J. Cancer*, **13**, 433.

BALDWIN, R. W. & PIMM, M. V. (1971) Influence of BCG Infection on Growth of 3-methylcholanthrene-induced Rat Sarcomas. *Eur. J. clin. biol. Res.*, **16**, 875.

BALDWIN, R. W. & PIMM, M. V. (1973*a*) BCG Immunotherapy of a Rat Sarcoma. *Br. J. Cancer*, **28**, 281.

BALDWIN, R. W. & PIMM, M. V. (1973*b*) BCG Immunotherapy of Local Subcutaneous Growths and Post-surgical Pulmonary Metastases of a Transplanted Rat Epithelioma of Spontaneous Origin. *Int. J. Cancer*, **12**, 420.

BALDWIN, R. W. & PIMM, M. V. (1973*c*) BCG Immunotherapy of Pulmonary Growths from Intravenously Transferred Rat Tumour Cells. *Br. J. Cancer*, **27**, 48.

BALDWIN, R. W. & PIMM, M. V. (1974) BCG Suppression of Pulmonary Metastases from Primary Rat Hepatomata. *Br. J. Cancer*, **30**, 473.

BALL, J. K. & MCCARTER, J. A. (1971) Effect of Polyinosinic-polycytidylic Acid on Induction of Primary or Transplanted Tumors by Chemical Carcinogen or Irradiation. *J. natn. Cancer Inst.*, **46**, 1009.

BANKS, G. T., BUCK, K. W., CHAIN, E. B., DARBYSHIRE, J. E. R. & HIMMELWEIT, F. (1969) Virus-like Particles in Penicillin Producing Strains of *Penicillium chrysogenum*. *Nature, Lond.*, **222**, 89.

BART, R. S. & KOPF, A. W. (1969) Inhibition of the Growth of Murine Malignant Melanoma with Synthetic Double-stranded Ribonucleic Acid. *Nature, Lond.*, **224**, 372.

BART, R. S., KOPF, A. W. & SILAGI, S. (1971) Inhibition of the Growth of Murine Malignant Melanoma by Polyinosinic-polycytidylic Acid. *J. invest. Derm.*, **56**, 33.

DE CLERCQ, E. & DE SOMER, P. (1971) Role of Interferon in the Protective Effect of the Double-stranded Polyribonucleotide against Murine Tumors Induced by Moloney Sarcoma Virus. *J. natn. Cancer Inst.*, **47**, 1345.

DE CLERCQ, E. & MERIGAN, T. C. (1971) Moloney Sarcoma Virus-induced Tumours in Mice. Inhibition or Stimulation by (PolyrI) (PolyrC). *Proc. Soc. exp. Biol. Med.*, **137**, 590.

ELMES, P. C. (1973) Therapeutic Openings in the Treatment of Mesothelioma. In *Biological Effects of Asbestos*. P. Borgorski, J. C. Gibson, V. Timbrell and J. C. Wagner (Eds). International Agency for Research on Cancer (Lyon). p. 277.

FISCHER, J. C., COOPERBRAND, S. R. & MANNICK, J. A. (1972) The Effects of Polyinosinic-polycytidylic Acid on the Immune Response of Mice to Antigenically Distinct Tumors. *Cancer Res.*, **32**, 889.

HEYES, J., CATHERALL, E. J. & HARNDEN, M. R. (1974) Antitumour Evaluation of a Ribonuclease Resistant Double-stranded RNA-polyquaternary Ammonium Complex (BRL 10739). *Eur. J. Cancer*, **10**, 431.

HOPPER, D. G., PIMM, M. V. & BALDWIN, R. W. (1975) Methanol Extraction Residue of BCG in the Treatment of Transplanted Rat Tumours. *Br. J. Cancer*, **31**, 176.

HOPPER, D. G., PIMM, M. V. & BALDWIN, R. W. (1976) Silica Abrogation of Mycobacterial Adjuvant Contact Suppression of Tumour Growth in Rats and Athymic Mice. *Cancer Immunol. Immunother.* In the press.

KREIDER, J. W. & BENJAMIN, S. A. (1972) Tumor Immunity and the Mechanism of Polyinosinic-polycytidylic Acid Inhibition of Tumor Growth. *J. natn. Cancer Inst.*, **49**, 1303.

LARSON, V. M., PANTELEAKIS, P. N. & HILLEMAN, M. R. (1970) Influence of Synthetic Double-stranded Ribonucleic Acid (Poly I:C) on SV40 Viral Oncogenesis and Transplant Tumour in Hamsters. *Proc. Soc. exp. Biol. Med.*, **133**, 14.

LEVY, H. B., LAW, L. W. & RABSON, A. S. (1969) Inhibition of Tumor Growth by Polyinosinic-polycytidylic Acid. *Proc. natn. Acad. Sci. U.S.A.*, **62**, 357.

MEIER, H., MYERS, D. D. & HUEBNER, R. J. (1970) Ineffectiveness of Poly I:C on Transplanted Tumours Induced by Methylcholanthrene. *Naturwissenschaften*, **57**, 248.

PARR, I., WHEELER, E. & ALEXANDER, P. (1973) Similarities of the Anti-tumour Actions of Endotoxin, Lipid A and Double-stranded RNA. *Br. J. Cancer*, **27**, 370.

PILCH, D. J. F. & PLANTEROSE, D. N. (1971) Effect on Friend Disease of Double-stranded RNA of Fungal Origin. *J. gen. Virol.*, **10**, 155.

PIMM, M. V. & BALDWIN, R. W. (1975) BCG Therapy of Pleural and Peritoneal Growth of Transplanted Rat Tumours. *Int. J. Cancer*, **15**, 260.

PIMM, M. V. & BALDWIN, R. W. (1976) Treatment of Transplanted Rat Tumours with Double-stranded RNA (BRL 5907). II. Treatment of Pleural and Peritoneal Tumours. *Br. J. Cancer*. **33**, 166.

PIMM, M. V. & HOPPER, D. G. (1975) Role of Immunocompetence in Localised BCG Suppression of Tumour Growth. *Br. J. Cancer*, (*Abstract*), **32**, 241.

PLANTEROSE, D. N., BIRCH, P. J., PILCH, D. J. F. & SHARPE, T. J. (1970) Antiviral Activity of Double-stranded RNA and Virus-like Particles from *Penicillium stolaniferum*. *Nature, Lond.*, **227**, 504.

PRICE, M. R. & BALDWIN, R. W. (1974) Immunogenic Properties of Rat Hepatomata Subcellular Fractions. *Br. J. Cancer*, **30**, 394.

SARMA, P. S., SHIU, G., NEUBAUER, R. H., BARON, S. & HUEBNER, R. J. (1969) Virus-induced Sarcoma of Mice: Inhibition by a Synthetic Polyribonucleotide Complex. *Proc. natn. Acad. Sci. U.S.A.*, **62**, 1046.

VANDEPUTTE, M., DATTA, S. K., BILLIAU, A. & DE SOMER, P. (1970) Inhibition of Polyoma-virus Oncogenesis in Rats by Polyriboinosinic-ribocytidylic acid. *Eur. J. Cancer*, **6**, 323.

WEXLER, H. (1966) Accurate Identification of Experimental Pulmonary Metastases. *J. natn. Cancer Inst.*, **36**, 641.

ZELEZNICK, L. D. & BHUYAN, B. K. (1969) Treatment of Leukemic (L-1210) Mice with Double-stranded Polyribonucleotides. *Proc. Soc. exp. Biol. Med.*, **130**, 126.